# Low GCNT2/I-Branching Glycan Expression Is Associated with Bladder Cancer Aggressiveness

**DOI:** 10.3390/biomedicines13030682

**Published:** 2025-03-10

**Authors:** Yuki Tobisawa, Keita Nakane, Takuya Koie, Tomoki Taniguchi, Masayuki Tomioka, Risa Tomioka-Inagawa, Kota Kawase, Makoto Kawase, Koji Iinuma

**Affiliations:** 1 Department of Urology, Graduate School of Medicine, Gifu University, Gifu 5011194, Japan; tobisawa.yuki.a7@f.gifu-u.ac.jp (Y.T.); nakane.keita.k2@f.gifu-u.ac.jp (K.N.); taniguchi.tomoki.a8@f.gifu-u.ac.jp (T.T.); tomioka.masayuki.e4@f.gifu-u.ac.jp (M.T.); inagawa.risa@gmail.com (R.T.-I.); kawase.kota.b5@f.gifu-u.ac.jp (K.K.); kawase.makoto.g5@f.gifu-u.ac.jp (M.K.); iinuma.koji.s0@f.gifu-u.ac.jp (K.I.); 2 Center for One Medicine Innovative Translational Research (COMIT), Institute for Advanced Study, Gifu University, Gifu 5011194, Japan

**Keywords:** glycan, GCNT2, I-antigen, bladder cancer, natural killer cell, tumor immunity

## Abstract

**Background/Objectives:** Abnormal glycan formation on the cancer cell surface plays a crucial role in regulating tumor functions in bladder cancer. In this study, we investigated the roles of glucosaminyl (*N*-acetyl) transferase 2 (GCNT2) in bladder cancer progression and immune evasion. GCNT2 synthesizes I-branched polylactosamine chains on cell surface glycoproteins. Understanding its functions will provide insights into tumor–immune interactions, facilitating the development of effective immunotherapeutic strategies. **Methods:** GCNT2 expression levels in bladder cancer cell lines and patient tumor samples were analyzed via quantitative polymerase chain reaction and immunohistochemistry. GCNT2 functions were assessed via overexpression and knockdown experiments. Its effect on natural killer (NK) cell-mediated cytotoxicity was evaluated via in vitro assay. Cytotoxic granule release from NK cells was measured via enzyme-linked immunosorbent assay. **Results:** GCNT2 expression was inversely correlated with bladder cancer aggressiveness in both cell lines and patient samples. Low GCNT2 levels were associated with advanced tumor stage and grade, suggesting the tumor-suppressive roles of GCNT2. Notably, GCNT2 overexpression enhanced the susceptibility of bladder cancer cells to NK cell-mediated killing, whereas its knockdown promoted immune evasion. GCNT2-overexpressing cells strongly induced the release of cytotoxic granules from NK cells, indicating enhanced immune recognition. **Conclusions:** Our findings suggest that aggressive bladder tumors evade NK cell immunity by decreasing the GCNT2 levels and that I-antigen glycans synthesized by GCNT2 are crucial for NK cell recognition by tumor cells. Our findings provide insights into the tumor–immune interactions in bladder cancer and GCNT2 and its associated pathways as potential targets for novel immunotherapeutic strategies.

## 1. Introduction

Cell surface carbohydrates significantly affect glycoprotein functions and play important roles in tumor cell proliferation and invasion [1,2,3]. Infiltration and dissemination of tumor cells from their primary location to distant sites are associated with unfavorable outcomes in several epithelial malignancies, including bladder cancer [4].

Recurrence and metastasis are the primary factors contributing to mortality in patients with bladder tumors. Notably, bladder tumors of identical grades and/or stages can exhibit varying rates of recurrence and progression [5]. Furthermore, specific molecular mechanisms underlying bladder tumor metastasis remain unclear.

**Figure 1:**
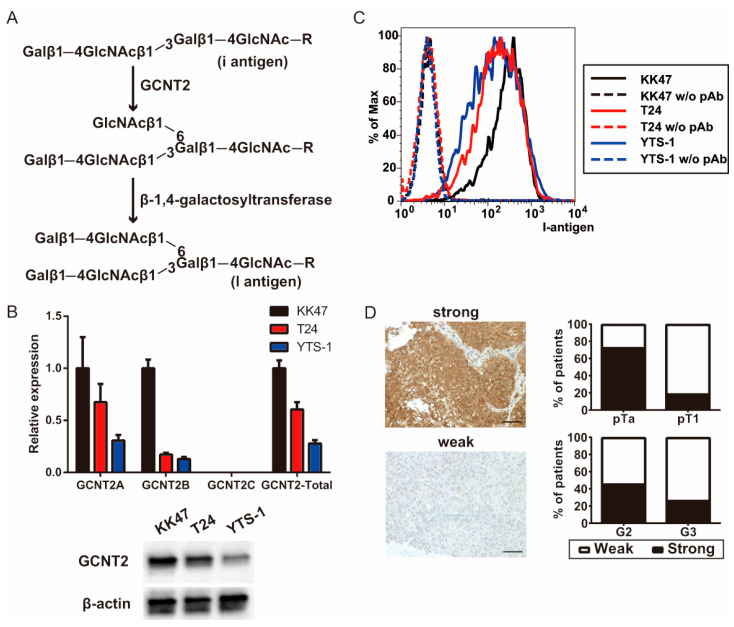
I-branching glucosaminyl (N-acetyl) transferase 2 (GCNT2) expression is correlated with bladder cancer malignancy. (**A**) Schema of the I-antigen biosynthetic pathways. (**B**) GCNT2 gene expression was measured via quantitative polymerase chain reaction (qPCR) analysis and protein expression was measured via western blotting analysis. (**C**) Cell surface I-antigen expression was determined using the anti-I antisera via flow cytometry. (**D**) Bladder cancer specimens were stained with the anti-GCNT2 antibodies, followed by staining with the horseradish peroxidase (HRP)-conjugated secondary antibodies. Counterstaining was performed using hematoxylin and eosin. GCNT2-positive cancer cells are indicated in brown. Column graph indicates the GCNT2 status and pathological parameters. Scale bars = 100 μm.

Glucosaminyl (*N*-acetyl) transferase 2 (GCNT2) [6,7,8], a key enzyme for I-branched poly-lactosamine chain (I-antigen) synthesis, functions by transferring *N*-acetylglucosamine from UDP-*N*-acetylglucosamine to the galactose of a linear lactosamine chain (I-antigen) via the beta-1, 6-linkage (Figure 1A). The transition from antigen i to antigen I during human development increases the number of polylactosamine chains and functional terminal structures [9], such as sialyl-Lewis X and sialyl-Lewis A [10]. Branched polylactosamine chains enhance the binding affinity of some lectins [11]. Alpha-1,6-mannosylglycoprotein 6-beta-*N*-acetylglucosaminyltransferase-mediated branched polylactosamine chains promote prostate cancer cell invasion [12]. GCNT1-mediated core2 branching *O*-glycans promote testicular tumor invasion and prostate cancer aggressiveness [13,14]. We previously showed a strong correlation between GCNT2 expression and prostate cancer malignancy [15]. Additionally, we reported that core2 branching *O*-glycans are involved in the malignant transformation of bladder cancer by regulating tumor immunity [16]. Considering the crucial roles of GCNT2 in glycan branching, we hypothesized that it affects the tumor cell dynamics, particularly in bladder cancer, in which cell surface glycosylation patterns are critical for tumor proliferation and invasion. To verify this, we aimed to explore the associations between GCNT2 expression and clinical features of patients with bladder cancer in this study.

We found an inverse correlation between GCNT2 expression in bladder cancer specimens obtained via transurethral resection of bladder tumor (TURBT) and cancer aggressiveness. Furthermore, GCNT2 overexpression in bladder cancer cells strongly stimulated the natural killer (NK) cell functions. Our findings indicate the crucial roles of GCNT2 in bladder cancer malignancy.

## 2. Materials and Methods

### 2.1. Materials

ISOGEN II Reagent was obtained from Nippon Gene (Tokyo, Japan). Horseradish peroxidase-conjugated goat anti-rabbit IgG (H + L) and anti-mouse IgG were purchased from Cell Signaling Technology (Danvers, MA, USA). Bovine serum albumin and 2-mercaptoethanol were acquired from Merck (Sigma-Aldrich, North Brunswick, NJ, USA). Tween 20 and penicillin/streptomycin solutions were obtained from FUJIFILM Wako Pure Chemical Corporation (Osaka, Japan). Precision Plus Protein Dual Color Standards were obtained from Bio-Rad (Tokyo, Japan). The *N*-glycosylation inhibitor tunycamycin was purchased from Wako Pure Chemical Corporation. The *O*-glycosylation inhibitor benzyl-alpha-GalNAc was purchased from Merck. The glucosylceramide synthetase inhibitor DL-threo-1-Phenyl-2-palmitoylamino-3-morpholino-1-propanol hydrochloride (PPMP) was purchased from Santa Cruz Biotechnology (Dallas, TX, USA).

### 2.2. Cells

KK-47 low-grade non-metastatic [17] and YTS-1 high-grade metastatic [18] bladder tumor cells were obtained from Dr. T Yoneyama at Hirosaki University (Hirosaki, Japan). T24 (high-grade) human urothelial carcinoma cell line was acquired from the American Type Culture Collection (Rockville, MD, USA). All cells were cultured in Roswell Park Memorial Institute (RPMI)-1640 medium (FUJIFILM Wako Pure Chemical Corporation) supplemented with 100 U/mL penicillin, 100 μg/mL streptomycin, and 10% fetal bovine serum (FBS; Biosera, Cholet, France).

### 2.3. Immunohistochemical Analysis of Bladder Tumor Specimens

The study cohort comprised 48 patients with bladder tumors who underwent TURBT at the Department of Urology, Gifu University Graduate School of Medicine, Gifu, Japan. Tumor specimens were formalin fixed and paraffin embedded. Deparaffinized specimens were incubated with the rabbit anti-human GCNT2 polyclonal antibodies (1:200, HPA026776; Merck), followed by incubation with the horseradish peroxidase-conjugated goat anti-mouse/rabbit IgG (Agilent, Santa Clara, CA, USA). Written informed consent was obtained from all patients prior to their participation in this study. The ethics committee of Gifu University approved the study protocol (study of the correlations between glycan-related gene expression patterns and cancer grades in urological cancers and cancer microenvironments; approval number: 2023-145). This study adhered to the Declaration of Helsinki.

### 2.4. Stable Transfection

YTS-1 cells were maintained in the RPMI-1640 medium supplemented with 10% FBS and seeded in a 35 mm cell culture dish (Corning, NY, USA) 24 h prior to transfection. Transfection was performed using the X-treme HP DNA transfection reagent (Roche Diagnostics, Basel, Switzerland) with 2 µg of the pEBmulti-neo GCNT2A or mock vector (FUJIFILM Wako Pure Chemical Corporation). Cell clones were selected in the RPMI-1640 medium supplemented with 10% FBS and 0.2 mg/mL G418 (Nacalai Tesque, Kyoto, Japan).

### 2.5. Determination of GCNT2 Levels in Bladder Cancer Cell Lines via Quantitative Real-Time Polymerase Chain Reaction (qPCR)

T24, KK47, and YTS-1 cells were cultured in 6-well plates (Corning) for two days. Total RNA was extracted from these cells using the ISOGEN II Reagent (Nippon Gene), and first-strand cDNA synthesis was performed using the ReverTra Ace qPCR RT Master Mix with gDNA Remover (ToYoBo, Osaka, Japan). Subsequently, qPCR analysis was performed using the GeneAce SYBR qPCR Mix α No ROX (Nippon Gene), according to the manufacturer’s protocol. All primer sequences have been previously reported [15]. Gene expression levels were normalized to those of human glyceraldehyde-3-phosphate dehydrogenase.

### 2.6. Flow Cytometry

Cells were cultured with DMSO, 2 mM BAG, 0.2 µg/mL tunycamycin, and 20 µg/mL PPMP on glass slides for 48 h at 80% confluence. Cells (1 × 10^5^) were incubated with or without primary antibodies in 100 mL of 1% bovine serum albumin-phosphate-buffered saline for 30 min on ice. After washing with phosphate-buffered saline, the cells were incubated with fluorescence-conjugated isotype-specific secondary antibodies and subjected to flow cytometry. Anti-I antigen human antiserum (1:1000; Ma) was used as the primary antibody. Human anti-I antiserum was generously provided by Dr. Eloise Giblett (Blood Bank Center, Seattle, WA, USA).

### 2.7. Western Blotting

Total cell lysates were prepared using 1% IGEPAL CA-630 with a protease inhibitor cocktail (Roche) and PhosStop (Roche), subjected to sodium dodecyl sulfate-polyacrylamide gel electrophoresis on a 4–15% gradient gel (Bio-Rad), and transferred onto a polyvinylidene difluoride membrane. Western blotting was performed using primary and horseradish peroxidase-conjugated secondary antibodies. Following incubation with the secondary antibodies, all samples were enzymatically detected using the Novex ECL Chemiluminescent Substrate Reagent Kit (Life Technologies, Tokyo, Japan) and visualized using the ChemiDoc XRS+ System (Bio-Rad). Protein expression was calculated using the signal intensity and normalized to that of human β-actin (GCNT2 intensity/β-actin intensity).

### 2.8. Cytotoxicity Assay

Primary NK cells isolated from the spleen of C57BL/6 mice using the Mojosort Mouse NK Cell Isolation Kit (BioLegend, San Diego, CA, USA) were cultured in the RPMI-1640 medium supplemented with 10% FBS and 1000 U/mL human recombinant IL-2 (PeproTech, Cranbury, NJ, USA) for five days and incubated with IL-2-activated NK cells at 37 °C for 4 h. Then, the release of lactate dehydrogenase from the lysed target cells was quantified. Percent cytotoxicity was calculated as follows: (experimental lactate dehydrogenase release − effector spontaneous release − target spontaneous release)/(target maximum release − target spontaneous release) × 100.

### 2.9. Matrigel Invasion Assay

Transwell cell culture chambers were used for the invasion assays. Cells (2.5 × 10^5^) were placed in the upper chamber, and 100 µg/mL fibronectin-RPMI1640 medium was added to the lower chamber. After 20 h of incubation, non-invading cells (upper side) were removed with a cotton swab, and invading cells (lower side) were fixed with methanol followed by Giemsa staining. The number of cells on the lower side was counted using a hybrid cell-counting system (Keyence, Osaka, Japan). These assays were conducted in triplicates.

### 2.10. Conjugation Formation Assay

Heterotypic cell conjugates were quantitatively determined using a double fluorescence assay [14] with some modifications. Bladder tumor target cells (2 × 10^6^ cells/mL) were stained with 5- or 6-(N-succinimidyloxycarbonyl)fluorescein 3′,6′-diacetate (CFSE) (Dojindo, Kumamoto, Japan). After 1 h of incubation of CFSE-labeled tumor cells with NK cells at 37 °C, cells were stained with PE-labeled anti-CD56 antibody, and the number of conjugates was counted using a FACSAria II (BD Biosciences, Franklin Lakes, NJ, USA). Double-colored (green and red) conjugates were calculated as the percentage of total CFSE-positive cells. 

### 2.11. Statistical Analyses

The chi-square test was used to analyze the associations between GCNT2 status and clinical and histopathological parameters of TURBT patients. Statistical analyses were conducted using the GraphPad Prism 9 software (GraphPad Software, San Diego, CA, USA).

## 3. Results

### 3.1. GCNT2 Expression in Inversely Correlated with Malignancy in Bladder Cancer

We examined GCNT2, an enzyme crucial for blood group I antigen formation, to verify its correlation with bladder cancer aggressiveness. qPCR analysis of bladder cancer cell lines revealed high GCNT2 levels in the non-invasive KK47 cells and low GCNT2 levels in the muscle-invasive T24 and YTS-1 cells (Figure 1B). Western blotting analysis also showed that the expression level of GCNT2 protein was 0.43 for T24 and 0.21 for YTS-1, based on KK47 (Figure 1B). Moreover, GCNT2 levels corresponded to the presence of I-antigen on the surfaces of bladder cancer cells (Figure 1C). The gating strategies are shown in Supplementary Figure S1. To assess the role of I-antigen expression in bladder tumor malignancy, immunohistochemical analysis of GCNT2 was conducted on TURBT specimens of 48 patients with bladder tumors using anti-GCNT2 antibodies. All patient characteristics are listed in Table 1. GCNT2-positive staining was observed in the cytoplasmic area near the nucleus, consistent with the known location of the Golgi complex. We found a significantly higher GCNT2 positivity rate in pTa (72.7%) than in pT1 (18.9%; chi-square test; *p* < 0.01). Furthermore, G2 tumors exhibited a higher GCNT2 positivity rate (45.8%) than the G3 tumors (26.1%; Figure 1D). These findings suggest an inverse relationship between GCNT2 expression and bladder cancer malignancy. Therefore, bladder tumors with low GCNT2 expression possibly develop highly malignant properties due to alterations in their cell surface carbohydrates.

### 3.2. Low GCNT2 Expression Facilitates Evasion from NK Cell Tumor Rejection Responses

**Figure 2:**
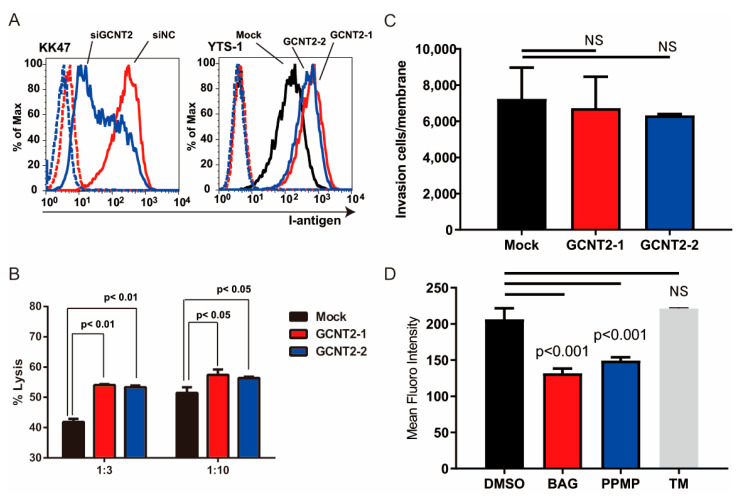
I-antigen regulates bladder cancer evasion from natural killer (NK) cells. (**A**) I-antigen expression was regulated by GCNT2. Dashed lines indicate the unstained control. (**B**) Cytotoxic effects of murine NK cells on YTS-1 and GCNT2-overexpressing YTS-1 (YTS1GCNT2-1 and 2) cells were assayed. (**C**) No significant differences in tumor cell invasion between YTS-1 and GCNT2-overexpressed YTS-1. (**D**) YTS-1 cells were cultured with the *O*-glycosylation inhibitor benzyl-alph-GalNAc (BAG), glucosylceramide synthetase inhibitor DL-threo-1-Phenyl-2-palmitoylamino-3-morpholino-1-propanol hydrochloride (PPMP), and *N*-glycosylation inhibitor tunycamycin (TM) for 48 h. I-antigen expression was determined by flow cytometry. BAG- and PPMP-treated cells showed a significant reduction in I-antigen levels. NS = not significant.

We have previously reported that branched glycans regulate tumor immunity [16] and invasiveness [15]. NK cells are crucial for the host’s defense against bloodstream tumors. The attack of NK cells on cancer cells is initiated by NK cell–cancer cell interactions mediated by the NK receptor–ligand interactions [19]. Therefore, we hypothesized that I-branching glycans regulate tumor immunity. To determine whether GCNT2 expression affects the NK cell–bladder cancer cell interactions, we compared NK immunity evasion between normal and GCNT2-overexpressing bladder cancer cells. We generated GCNT2-overexpressing YTS-1 (YTS1-GCNT2) cells and temporarily suppressed GCNT2 expression in KK47 cells (KK47siGCNT2) via transfection (Supplementary Figure S2). Flow cytometric analysis of I-antigen expression on the cell surface revealed a correlation between the I-antigen and GCNT2 levels (Figure 2A). Subsequently, we assessed NK cell cytotoxicity in bladder tumor cells. Mouse NK cells eliminated the GCNT2-overexpressing YTS1-GCNT2 cells more efficiently than the GCNT2-normally expressing YTS-1 cells at various effector–target ratios (Figure 2B). Because tumor invasiveness also regulates tumor malignancy, we tested whether GCNT2 regulates bladder cancer invasiveness. Matrigel invasion assay revealed no significant differences between YTS1 and YTS1-GCNT2 (Figure 2C). I-antigens potentially form *O*-glycans, *N*-glycans, and Glycolipids [15]. Therefore, we confirmed that GCNT2 acts on glycans in bladder cancer cells. YTS-1 cells were treated with benzyl-alpha-GalNAc (BAG), tunicamycin (TM), and DL-threo-1-Phenyl-2-palmitoylamino-3-morpholino-1-propanol hydrochloride (PPMP). The treated cells were assessed by flow cytometry using anti-I antisera. PPMP and BAG treatment decreased I-antigen expression (Figure 2D). In contrast, TM treatment did not change I-antigen expression. These results suggest that the I-antigen formed on the *O*-glycan and glycolipid in bladder cancer cells. Our findings suggest that tumor cells with low GCNT2 levels have decreased surface expression of I-antigen on *O*-glycans and glycolipids possess an enhanced capacity to evade NK cell-mediated immunity.

### 3.3. Effects of I-Branching Glycan on NK Cell Functions

**Figure 3:**
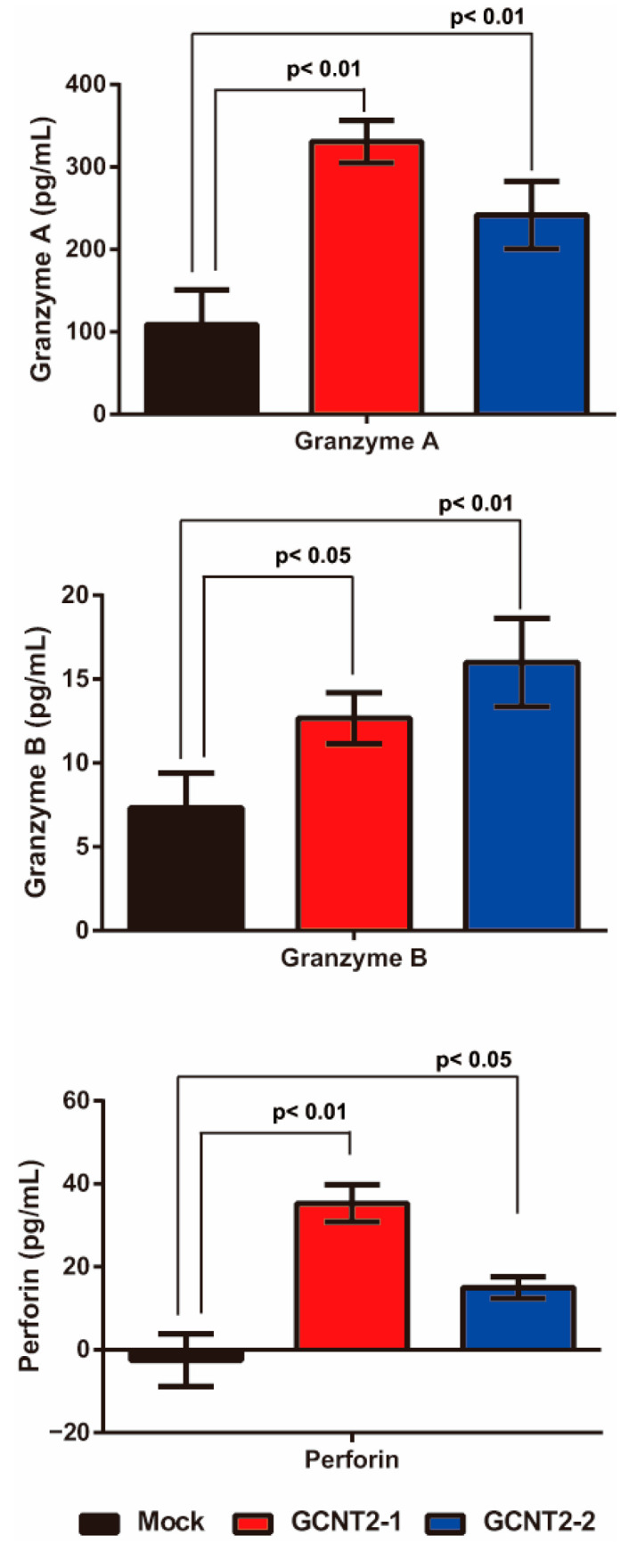
Effects of I-antigen on NK cell functions. Cytotoxic granule release from NK cells co-cultured with the YTS-1 and YTS1GCNT2-1 and 2 cells was analyzed. All assays were conducted in triplicate.

NK cells are activated via interactions with target cells and subsequently release granule-containing substances to induce target cell death. These granular molecules include granzyme A, granzyme B, and perforin, which trigger apoptosis in the target cells. First, we confirmed whether I-branching glycans suppress NK cell–tumor cell interactions. The NK cell-tumor cell conjugation assay revealed no significant differences in YTS-1 and YTS1-GCNT2 (Supplementary Figure S3). Next, we evaluated the release of these molecules via NK cell–bladder cancer cell interactions by analyzing the co-culture supernatant via enzyme-linked immunosorbent assay. YTS-1 cells with high GCNT2 levels exhibited significantly greater secretion of granzyme A, granzyme B, and perforin than those with normal GCNT2 levels (Figure 3). These observations suggest that low I-antigen levels reduce the interactions between NK and bladder cancer cells, thereby suppressing the release of granzyme A, granzyme B, and perforin.

## 4. Discussion

This study revealed the following key insights into GCNT2 expression and its relationship with bladder cancer malignancy: (1) GCNT2 expression was inversely correlated with bladder cancer aggressiveness, (2) GCNT2 expression affected the NK cell-mediated immunity in bladder cancer cells, and (3) I-branching glycans regulated by GCNT2 impacted the NK cell functions. These findings suggest that GCNT2 plays an important role in bladder cancer malignancy, possibly by modulating the tumor cell interactions with NK cells. Low GCNT2 levels in aggressive tumors are possibly a mechanism to evade the NK cell-mediated immune responses.

GCNT2 is a key enzyme in I-antigen synthesis. We have previously reported that I-branching glycans regulate prostate cancer invasiveness by enhancing integrin signaling [15]. In breast cancer, GCNT2 is also associated with invasiveness by regulating epithelial-to-mesenchymal transition [20]. Nakamura et al. reported that GCNT2 expression is regulated by DNA methylation, and hypomethylation of the GCNT2 promoter which is closely associated with lymph node metastasis and poor prognosis [21]. Gene expression, including that of GCNT2, is silenced by DNA methylation, and DNA hypomethylation of GCNT2 increases its expression [21]. Therefore, in these cancer types, increased GCNT2 expression is associated with tumor cell invasiveness and malignancy. In the present study, cell motility was not found to be regulated by I-antigen expression in bladder cancer cells. Therefore, we expected that the glycans modified by GCNT2 would be different; however, the results showed that the I-antigen was also formed on *O*-glycans and glycolipids in bladder cancer, similar to prostate cancer [15]. The cause of these differences is not clear but may be due to differences in the underlying cellular systems, as prostate and breast cancers are adenocarcinomas, whereas bladder cancer is a transitional cell carcinoma. Further studies are required to confirm this mechanism.

Our data revealed that GCNT2 downregulation was beneficial for bladder cancer aggressiveness. Sexton et al. showed lower GCNT2 expression in diffuse and intestinal-type gastric carcinomas than in normal tissue [22]. Dong et al. also showed that low levels of GCNT2 expression are relevant for disease progression in cutaneous and adult T-cell lymphomas [23]. Sweeney et al. reported that, in melanoma cells, GCNT2 was inversely correlated with clinical progression [24]. They found that melanomas exhibit significant transcriptional changes in glycosylation-related genes, and GCNT2 is one of these signatures. In melanoma cells, loss of GCNT2 leads to melanoma progression, tumor cell growth, and survival [24]. Loss of GCNT2 leads to downregulation of I-branching glycan, enhancement of IGF-1 binding to its receptor, or acceleration of extracellular matrix-integrin interaction [24,25,26]. Our results suggest that GCNT2 does not affect integrin-mediated pathways in bladder cancer cells at least, since the expression level of GCNT2 did not affect cell invasiveness or intercellular adhesion, which are likely to be mediated by integrins.

NK cells play crucial roles in the innate immune responses against cancer, serving as the first line of defense against tumors and metastases [27,28]. They recognize and eliminate transformed cells while sparing healthy cells because of their germline-encoded receptors [27]. They kill target cells non-specifically and independently of antibodies or prior activation, making them particularly effective against circulating tumor cells and some hematological cancers such as acute myeloid leukemia [27,28]. We previously reported that branched glycans formed by GCNT1 regulate the NK cell–tumor cell interactions [11,14]. In this study, I-branching glycans stimulated immune activation receptors in NK cells. I-branching glycan leads to an increasingly functional terminal structure, such as sialyl-Lewis X [10]. Sialyl-Lewis X-overexpressing tumor cells are potently eliminated by NK cells [29]. Therefore, in bladder cancer, low I-antigen levels decrease the functional glycan levels, facilitating NK cell evasion. Overall, this study highlights the impact of branched carbohydrates on bladder tumor metastasis. Future studies should explore the carbohydrates on tumor cell surfaces to elucidate the complex mechanisms underlying tumor metastasis.

## Figures and Tables

**Table 1:** Characteristics of patients who underwent the transurethral resection of bladder tumor.

Age (Mean [Range])	67	(47–83)	
Gender	Male Female	40 8	(83%) (17%)
Pathological stage	pTa pT1	11 37	(23%) (77%)
Grade	G2 G3	24 24	(50%) (50%)
Histological type	UC	48	(100%)

## Data Availability

The data presented in this study are available upon request from the corresponding author. The data are not publicly available due to privacy and ethical reasons.

## Supplementary Materials

The following supporting information can be downloaded at https://www.mdpi.com/article/10.3390/biomedicines13030682/s1, Figure S1: Gating strategy of I-branching Glycan expression; Figure S2: GCNT2 knockdown efficiency in KK47; Figure S3: Conjugation assay Natural killer (NK) cell-YTS-1 (Mock) or YTS1GCNT2 (GCNT2-1).
