# Supplementary material for: Low GCNT2/I-Branching Glycan Expression Is Associated with Bladder Cancer Aggressiveness
Source: Biomedicines. 2025 Mar 10;13(3):682. doi: 10.3390/biomedicines13030682 (PMC11940493; doi:10.3390/biomedicines13030682)
Supplement: Supplementary file 1 [file biomedicines-13-00682-s001.zip › biomedicines-3512292-supplementary.pdf]

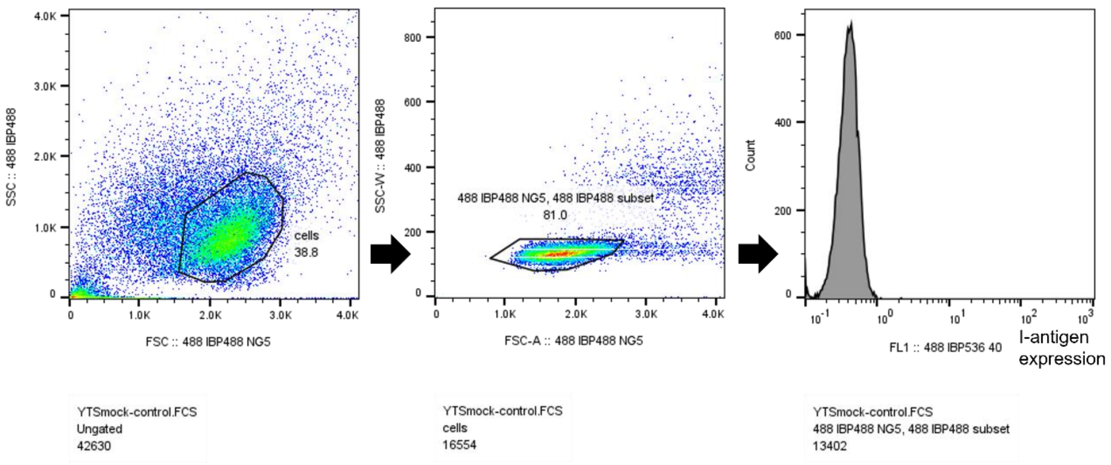

Supplementary Figure S1

Gating strategy of I-branching Glycan expression

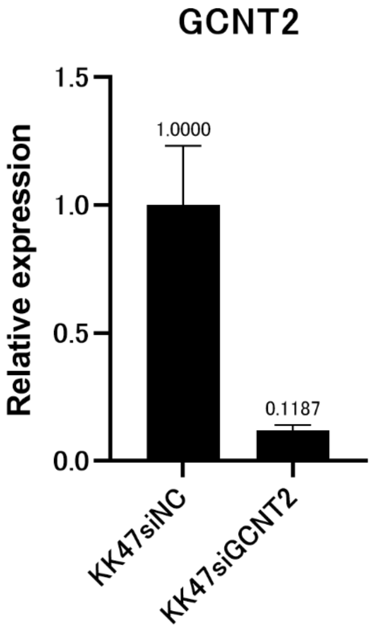

Supplementary Figure S2

GCNT2 knockdown efficiency in KK47

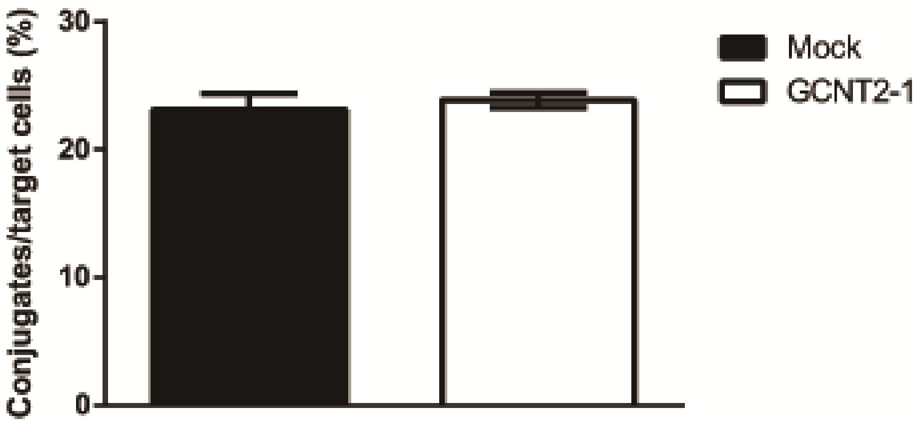

Supplementary Figure S3

Conjugation assay Natural killer (NK) cell-YTS-1 (Mock) or YTS1GCNT2 (GCNT2-1)
